# Current statins show calcium channel blocking activity through voltage gated channels

**DOI:** 10.1186/s40360-016-0086-5

**Published:** 2016-09-21

**Authors:** Niaz Ali, Robina Begum, Muhammad Saleh Faisal, Aslam Khan, Muhammad Nabi, Gulfam Shehzadi, Shakir Ullah, Waqar Ali

**Affiliations:** 1 Department of Pharmacology, Institute of Basic Medical Sciences (IBMS), Khyber Medical University, Peshawar, Pakistan; 2 Department of Pharmacy, Abasyn University, Peshawar, Khyber Pakhtunkhwa Pakistan

**Keywords:** Statins, Simvastatin, Atorvastatin, Fluvastatin, Rosuvastatin, Voltage gated calcium channels, Verapamil

## Abstract

**Background:**

Statins are used for treatment of hypercholestremia. Common adverse reports associated with use of statins are generalized bodyache, rhabdomyolysis, muscles weakness and gastrointestinal disorders. The current work is an attempt to explain how smooth muscles of gastrointestinal tissues are affected by the current statins (Simvastatin, atorvastatin, fluvastatin and rosuvastatin).

**Methods:**

Effects of the current statins were studied on spontaneous activity of isolated rabbits’ jejunal preparations. Different molar concentrations (10^−12^–10^−2^M) of the statins were applied on spontaneously contracting rabbits’ jejunal preparations. As statins relaxed spontaneous activity, so we tested the statins on KCl (80 mM) induced contractions in similar test concentrations. Positive relaxant statins were tested again through construction of Calcium Concentration Response Curves (CCRCs) in the absence and presence of the statins using verapamil, a standard calcium channel blocker. CCRCs of statins were compared with CCRCs of verapamil.

**Results:**

Simvastatin, atorvastatin, fluvastatin and rosuvastatin relaxed the spontaneous and KCl-induced contractions. IC_50_ for simvastatin on spontaneous rabbit’s jejunal preparations is −5.08 ± 0.1 Log 10 M. Similarly, IC_50_ for KCl-induced contractions is −4.25 ± 0.01 Log 10 M. Mean IC_50_ (Log 10 M) for atorvastatin on spontaneous rabbit’s jejunal preparations and KCl-induced contractions are −5.19 ± 0.07 and −4.37 ± 0.09, respectively. Fluvastatin relaxed spontaneous activity of rabbits’ jejunal preparations with an IC_50_ (Log 10 M) −4.5 ± 0.03. Rosuvastatin relaxed spontaneous as well as KCl (80 mM) induced contractions with respective IC_50_ (Log 10 M) −3.62 ± 0.04 and −4.57 ± 0.06. In case of CCRCs, tissues pre-treated with 4.6 μg/ml of simvastatin, have IC_50_ = −1.84 ± 0.03 [log (Ca^++^) M] vs control IC_50_ = −2.54 ± 0.04 [log (Ca^++^) M]. Similarly, atorvastatin, fluvastatin and rosuvastatin produced significant right shift in IC_50_ for CCRCs (P ≤ 0.05). In case of verapamil, IC_50_ for control curves is −2.45 ± 0.06 [log (Ca ^++^) M], while IC_50_ in presence of verapamil (0.1 μM) is −1.69 ± 0.05 [log (Ca ^++^) M]. Statins produced right shift in the IC_50_ of CCRCs_._ The effects of statins are like that of effects of verapamil, a standard calcium channel blocker.

**Conclusions:**

Our findings suggest that current statins have calcium antagonistic effects that act on voltage gated calcium channels that may provide a rationale for cause muscle weakness and gastrointestinal disorders.

## Background

There are very hot discussions about the safety profile and role of statins use in low risk cardiovascular patients. Even stake holders in health professions ask for an impartial review of statins especially in patients who are at low risk [1]. Statins are therapeutically effective lipid lowering drugs, which are used to reduce blood cholesterol in patients with hypercholestremia [2]. Statins reduce the development of atherosclerosis and prevent formation of atheromatous lesion [3]. Statins inhibit the enzyme HMG-CoA reductase and prevents the *de novo* synthesis of cholesterol as well [4]. Statins decrease intracellular cholesterol level, which increases the LDL receptors that can combine and internalize circulating LDLs. Consequently plasma cholesterol level is reduced by inhibiting cholesterol synthesis and raise catabolism of LDLs [5]. It is explicit that statins decrease cholesterol in addition to have pleotropic action [6]. Three lipid lowering drugs i.e. lovastatin, simvastatin and pravaststin were approved for marketing in 1990 [7, 8]. Statins valuable effects were seen in cardiac patients [9]. With passage of time, its adverse effects started appearing. Following administration of normal doses, statins are quickly absorbed and gain peak plasma concentrations within 4 h. They are metabolised by Cytochrome P450, which is composed of 30 isoenzymes. [10, 11]. Fluvastatin is metabolized through CYP2C9 and drugs like fluconazole and diclofenac (inhibitors of CYP2C9) interact to raise plasma levels of the statins [12]. Common adverse effects of statins are muscle pain and weakness, which can progress to rhabdomyolysis [13, 14]. Myositis, myalgia and cataract [15] are mostly caused by fluvastatin and simvastatin. Cerivastatin was withdrawn from market because of myotoxicity produced in most of the patients [16]. Most common unwanted effects of statin on gastrointestinal system are constipation, dyspepsia, abdominal pain, nausea, vomiting, heartburn and flatulence [15]. Smooth muscles’ contraction of GIT is due to calcium influx, which is regulated by calmodulin mechanism. Stimulating fibres in myosin filaments cause to develop attractive forces between actin and myosin filaments which is responsible for smooth muscles contractions. Total cholesterol level in human body is decreased by regulating cholesterol quantity in intestinal wall due to reducing Acetyl Co acyltransferase enzyme [17]. Statins also deregulate calcium channels that stimulate differentiated phenotype of vascular smooth muscle cells, as a consequence, reactivate calcium influx pathway and also upregulate L-type calcium channels, where calcium channel blocker effect is synergized in vascular cells [18]. But this upregulation takes time to develop and was studied in cell lines. It was also observed in animal study that using long term statins in hypertensive animals normalized blood pressure and wall of blood vessels [19]. Perhaps, this may be attributed to reports which say that smooth muscles’ cell migration, proliferation and invasion was inhibited by statins due to preventing isoprenoid pathways that subsequently inhibit Rhoprenylation of smooth muscles’ cells [20]. As there are reports for GI upsets with statins in start of therapy, hence we designed current study to test the direct effects of statins on isolated rabbits’ jejunal preparations. Thus our objective was to check the current statins for possible inhibitory effects on voltage gated calcium channels that may describe possible rationale for gastrointestinal disorders.

### Study setting

The study was carried out in Department of Pharmacology, Institute of Basic Medical Sciences, Khyber Medical University, Peshawar, Khyber Pakhtunkhwa, Pakistan.

## Methods

### Effects of current statins on isolated rabbits’ jejunal preparation

The aim of the current work is to find out possible effects of some current statins (simvastatin, atorvastatin, fluvastatin and rosuvastatin) on isolated rabbits’ jejunal preparations.

### Drugs and standards

Analytical grade chemicals were used in the experiments. Acetylcholine was purchased from BDH, Poole, England, which was used for maintenance of isolated tissues. Raw materials of rosuvastatin and atorvastatin as calcium salts were obtained from Ferozsons Laboratories Pvt. Ltd. Nowshera, Pakistan. Raw material of simvastatin was taken from Polyfine Pharmaceutical Industry, Peshawar. Fluvastatin of Novartis Pharma were purchased from local market of Peshawar, Pakistan. Raw materials, which had poor solubility in Tyrode’s solutions, were suspended in 0.01 % Carboxy Methyl Cellulose (CMC). However, a negative control of 0.01 % of CMC in deionized water was run to rule out any possible effects of CMC. All solutions and suspensions were freshly prepared on the same days of experiments.

### Animals

Local breed rabbits weighing (1.5–2.0 kg; either sex) were used in the experiments. They were kept in animal house under controlled environment at Institute of Basic Medical Sciences, Khyber Medical University, Peshawar, Pakistan. The animals ware fasted overnight before the days of experiments. They had free access to water. The study protocols were approved by the Advanced Study & Research Board and Ethical Board (Approval No. Dir/KMU/-EB/SE/000138) of the Khyber Medical University, Peshawar, Pakistan.

### Data recording

Intestinal responses were recorded using Force Transducer (Model No: MLT 0210/A Pan Lab S.I), connected through an amplifier FE 221 attached with four channels Power Lab (Model No: 4/25 T) AD Instruments, Australia. Lab Chart 7 was used to record and interpret the intestinal responses of isolated jejunal preparations.

### Physiological solutions used in the experiments

Normal Tyrode’s solution, Potassium Normal (Ca^++^ free) Tyrode’s solution and Potassium Rich (Ca^++^ free) Tyrode’s solutions were used in the experiments. All solutions were prepared in deionized water on the same day of experiments.

### Effects of statins on spontaneous rabbits’ jejunal preparations

Abdomens of overnight fasted rabbits were opened. Their jejunums were removed and placed in petri dishes containing Tyrode’s solution. The tissues were maintained with constant supply of carbogen gas (95 % O_2_, 5 % CO_2_) [21]. Pieces of about 1.5 cm were cut from the jejunums and mounted in organ bath containing Tyrode’s solution already maintained with carbogen gas (95 % O_2_, 5 % CO_2_) on 37 ± 1 °C. The composition of Tyrode’s solution was (mM) KCl 2.7, NaH_2_PO_4_ 0.4, NaCl 136.9, Glucose 5.6, MgCl_2_ 1.1, CaCl_2_ 1.8, NaHCO_3_ 11.9 on _p_H 7.4. Test concentrations of current statins were prepared in deionized water as mentioned above. The statins were added in cumulative manner to the organ bath in test concentrations Log10 (1 × 10^−12^–1 × 10^−3^) M which entail their plasma levels. The test samples were applied in a period of 1 min gap. Effects on spontaneous jejunal preparations were recorded as per our reported procedures [22, 23]. Earlier, tissues were stabilized for a period of 30 min before testing the statins.

### Effects of statins on KCl (80 mM)-induced contractions

As KCl-depolarizes the tissues and keep the tissues in sustained contractions, hence any relaxing effects are usually, but not necessarily, regarded as to follow voltage gated calcium channels. So we tested the statins on KCl-induced contraction in similar equimolar concentrations Log10 (1 × 10^−12^–1 × 10^−3^) M in cumulative manners. Their effects are recorded [22, 24]. IC_50_ were calculated using Graph Pad Prism.

### Effects of statins on calcium concentration response curves (CCRCs)

For confirmation of involvement of voltage gated calcium channels, we constructed CCRCs in a range of calcium concentration 1 × 10^−4^–256 × 10^−4^ M in absence and presence of different concentrations of statins as per our reported practice [23, 25, 26]. Verapamil was used as standard calcium channel blocker [23, 25–27]. Briefly describing the procedure, the tissues were maintained in Tyrode’s solution. After stabilization of tissues, the tissues were exposed to a series of wash with Tyrode’s Normal (calcium free) solution followed by exposure to K-Rich Tyrode’s solution. K-Rich Tyrode’s solution composition was (mM) NaCl 91.04, KCl 50, NaH_2_ PO_4_ 0.42, MgCl_2_ 1.05, EDTA 0.1, NaHCO_3_ 11.90 and glucose 5.55. This lead to decalcification of tissues. The temperature was maintained on 37 ± 1 °C. Control CCRCs were constructed in absence of statins. Then CCRCs were constructed in presence of different concentrations of statins following an incubation period of 1 h. Similarly, curves were constructed in absence and presence of verapamil, a standard calcium channel blocker. The CCRCs were compared for any possible right shift.

### Statistical analysis

Effects of test concentrations of statins on isolated rabbits’ jejunal preparations were plotted versus respective concentrations of statins as dose repose curves using Graph Pad Prism. Effects were expressed as % of control maximum for spontaneous as well as KCl-induced contractions. For CCRCs, control curves were drawn in Graph Pad Prism. CCRCs were also drawn in presence of respective statins concentrations. Data were analysed using nonlinear regression (curve fit) method with built in equation for sigmoidal dose response using Graph Pad Prism. Two way ANNOVA was used to determine the significances of concentration versus responses at 95 % CI with P < 0.05.

## Results

**Fig. 1:**
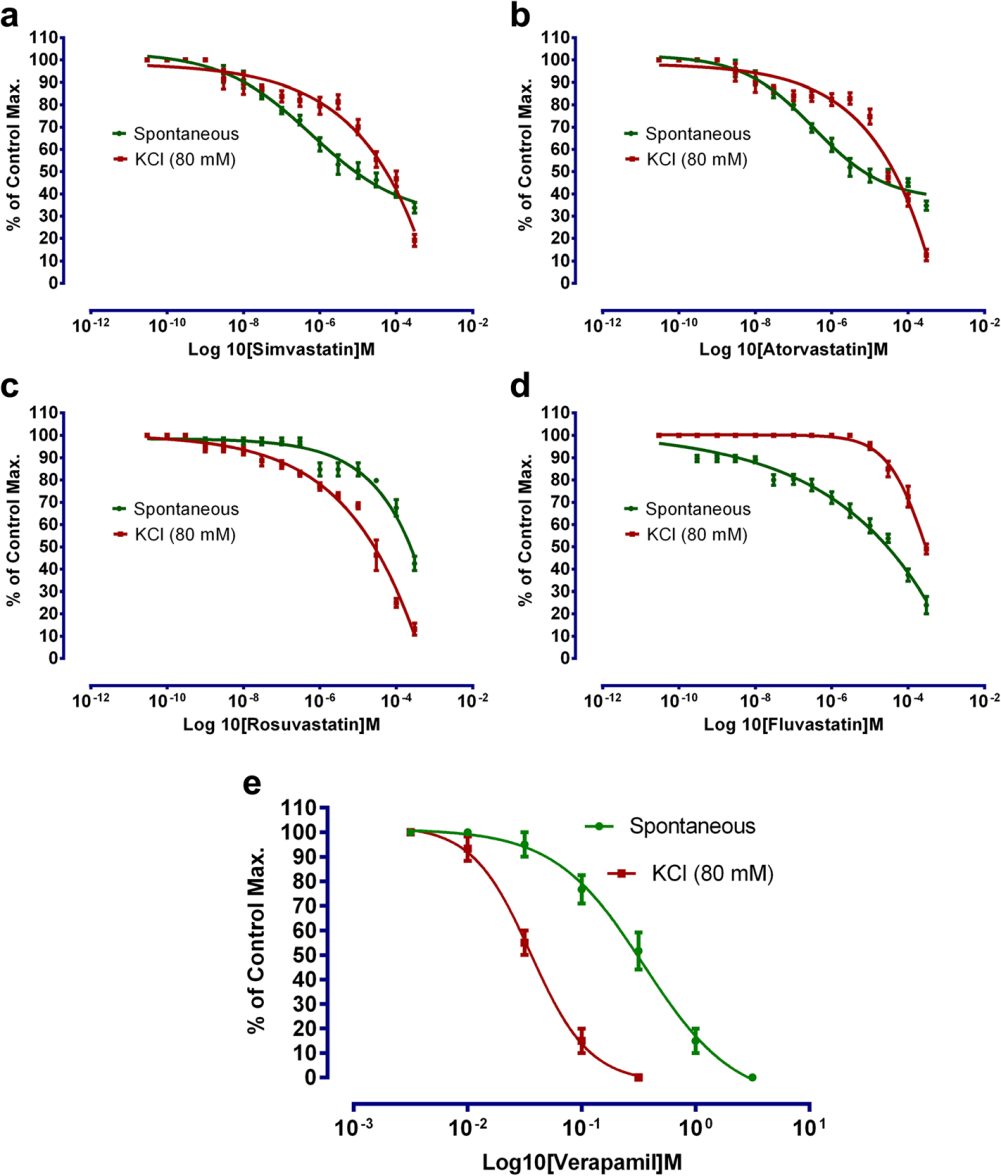
To show the effect of **a** simvastatin, **b** atorvastatin, **c** rosuvastatin, **d** fluvastatin, and **e** verapamil on spontaneous and KCl-induced contractions. (Effects is expressed as percent of control maximum, *n* = 5)

Effects of statins on spontaneous and KCl-induced contractions are shown in Fig. 1. Effects of simvastatin on spontaneous activity of rabbits’ jejunal preparations and KCl (80 mM) induced contractions are shown in Fig. 1a. Decrease in spontaneous activity is evident on concentration −6.15 Log 10[Simvastatin] M. IC_50_ value on spontaneous rabbits’ jejunal preparations is −5.08 ± 0.1 Log 10[Simvastatin] M. Relaxant effects of simvastatin was maximum (65 %) of control maximum on concentration −3.4 Log10[Simvastatin] M. Similarly, KCl-induced contractions were relaxed in concentration −5.2 Log 10[Simvastatin]M. Mean IC_50_ value of simvastatin for KCl induced contraction Log 10[Simvastatin] M was −4.25 ± 0.01 (Table 1). Similarly, atorvastatin effects on spontaneous activity of rabbits’ jejunal preparations and KCl (80 mM) induced contractions are shown in Fig. 1b. Decrease in spontaneous activity was evident on concentration −4.3 Log[Atorvastatin] M. Mean IC_50_ value on spontaneous rabbit’s jejunal preparations and KCl-induced contractions are −5.19 ± 0.07 Log 10[atorvastatin] M and −4.37 ± 0.09 Log 10[atorvastatin]M. Relaxant effect on KCl induced contractions was maximum (70 %) in concentration −3.3 Log 10[atorvastatin] M.

**Table 1:** To show the effects of current statins on spontaneous and 80mM KCl-induced contractions in isolated rabbits’ jejunal preparations (*n* = 5)

Types of contractions	Mean Log Molar IC_50_ of statins (Mean ± SD)				
	Simvastatin	Atorvastatin	Rosuvastatin	Fluvastatin	Verapamil
Spontaneous contractions	−5.08 ± 0.1	−5.19 ± 0.07	−3.62 ± 0.04	−4.5 ± 0.03	−0.5 ± 0.01
KCl-induced contractions	−4.25 ± 0.01	−4.37 ± 0.09	−4.57 ± 0.06	−3.52 ± 0.02	−1.43 ± 0.04

Fluvastatin effects on spontaneous activity of rabbits’ jejunal preparations and KCl (80 mM) induced contractions are shown in Fig. 1d with respective mean IC_50_ values of −3.63 ± 0.04 Log 10[fluvastatin] M and −3.52 ± 0.02. Spasmolytic effect of fluvastatin was maximum on concentration −3.3 Log 10[fluvastatin]M. Rosuvastatin relaxed spontaneous as well as KCl (80 mM) induced contractions with respective mean IC_50_ (Log 10[rosuvastatin]M) of −3.62 ± 0.04 and −4.57 ± 0.06 Fig. 1c. Decrease in spontaneous activity started in concentration −5.2 Log 10[rosuvastatin]M. Maximum relaxing effects on spontaneous and KCl-induced contractions were respectively 70 and 40 % on −3.39 Log 10[rosuvastatin]M. Verapamil relaxed both spontaneous and KCl-induced contractions with respective IC_50_ (Log 10[Verapamil]M) values of −0.5 ± 0.01 and −1.43 ± 0.04 (Fig. 1e).

### Calcium channel blocking activity on Rabbits’ jejunal preparations

Contractions in smooth muscles occur due to calcium influx. Two types of Ca^2+^ channel are involved i.e. voltage dependent and receptor-linked Ca^2+^channels. Intestinal contractions are due to cytosolic free calcium levels which passes through voltage gated calcium channels in sarcoplasmic reticulum [21, 25, 28, 29]. It has been reported that KCl opens the voltage gated calcium channels. Thus an agent which relaxes the KCl-induced contractions is considered to have calcium channel blocking activity. But as reported that positive relaxing effects on KCl-induced contraptions not always follow inhibition of voltage gated calcium channels, hence, construction of calcium concentration response curves will testify with a right shift in the tissues or otherwise [30].

**Fig. 2:**
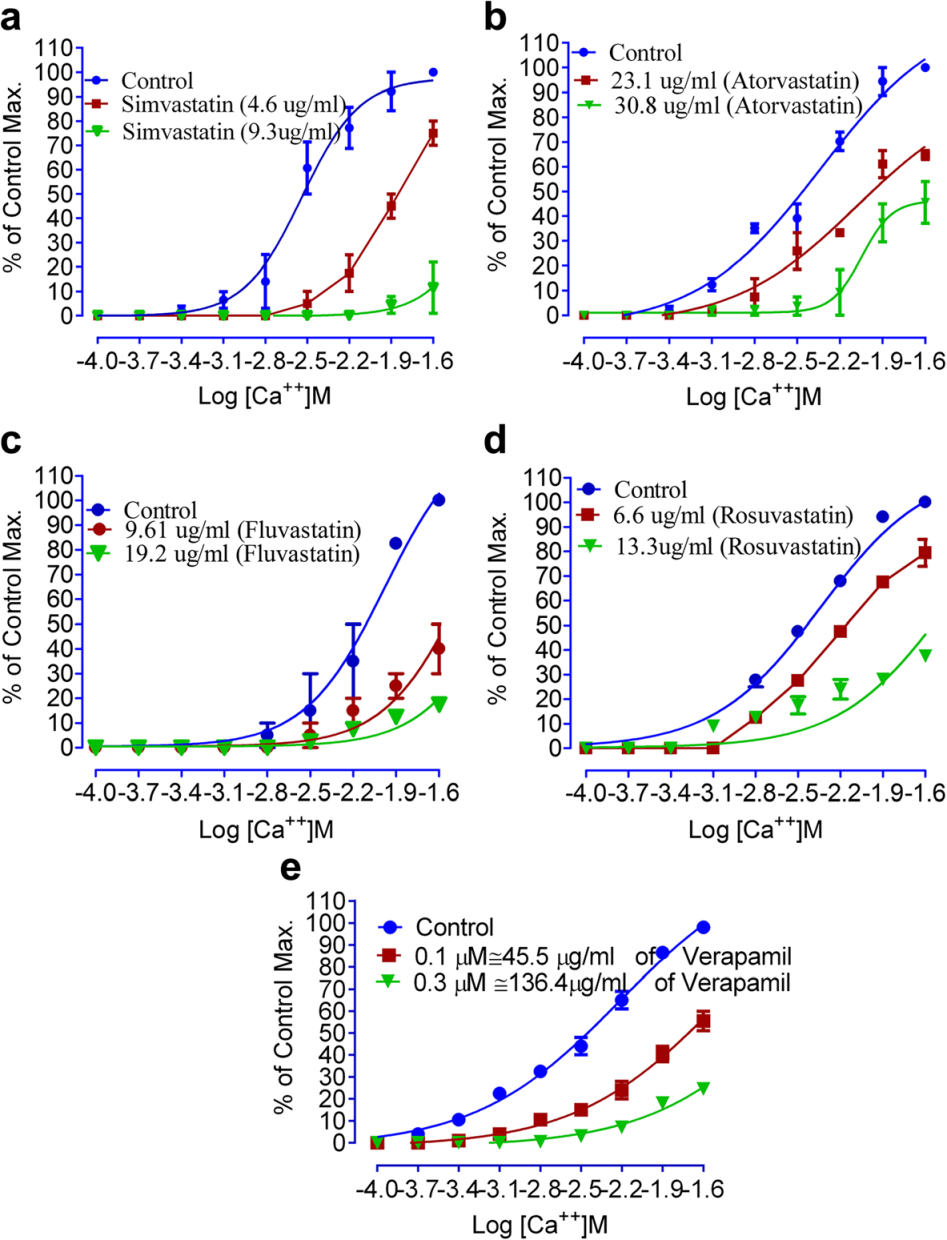
To show the effect of **a** simvastatin, **b** atorvastatin, **c** fluvastatin, **d** rosuvastatin, and **e** verapamil on calcium chloride curve compared to that of the respective controls

As statins (simvastatin, atorvastatin, fluvastatin and rosuvastatin) in different concentrations showed relaxing properties both on spontaneous and KCl-induced contractions, hence, we constructed CCRCs. Calcium chloride curves in the absence (control) and presence of test samples of simvastatin, atorvastatin, fluvastatin and rosuvastatin are shown in Fig. 2. According to Fig. 2a, IC_50_ for control curve, in case of simvastatin, is −2.54 ± 0.04 [log (Ca ^++^) M]. While tissues pre-treated with 4.6 μg/ml of simvastatin, have IC_50_ = −1.84 ± 0.03 [log (Ca ^++^) M]. Similarly, IC_50_ for atorvastatin, control curve is−2.48 ± 0.06 [log (Ca ^++^) M]. In presence of 23.1 μg/ml of atorvastatin, IC_50_ = −1.99 ± 0.04 [log (Ca ^++^) M] (Fig. 2b). IC_50_ ([log (Ca ^++^) M]) value for control and in presence of 9.6 μg/ml of fluvastatin are −2.11 ± 0.05 and −1.56 ± 0.03, respectively (Fig. 2c). In presence of rosuvastatin 6.6 μg/ml value of IC_50_ = −2.17 ± 0.03, while for control IC_50_ = −2.44 ± 0.03 [log (Ca ^++^) M] Fig. 2d. IC_50_ values are shown in Table 2. In case of verapamil, IC_50_ [log (Ca ^++^) M] for control curves is −2.45 ± 0.06, while IC_50_ in presence of verapamil (0.1 μM = 45.46 μg/ml) is −1.69 ± 0.05 [log (Ca ^++^) M] (Fig. 2e). It is evident from Fig. 2 and Table 2 that all statins produced a right shift in IC_50_ for CCRCs_._ The effects of statins are like that of effects of verapamil, a standard calcium channel blocker with a right shift [22, 23, 25].

**Table 2:** To represent the IC_50_ values in absence (control) and presence (test concentrations) of test statins

Statins	CCRCs specifications	IC_50_ Log [Ca^++^]M
Simvastatin	Control	−2.54 ± 0.04
	Test concentration 4.6 μg/ml	−1.84 ± 0.03^**^
Atorvastatin	Control	−2.48 ± 0.06
	Test concentration 23.1 μg/ml	−1.99 ± 0.04^**^
	Test concentration 30.8 μg/ml	−1.68 ± 0.03^**^
Fluvastatin	Control	−2.11 ± 0.05
	Test concentration 9.61 μg/ml	−1.56 ± 0.03^**^
Rosuvastatin	Control	−2.44 ± 0.03
	Test concentration 6.6 μg/ml	−2.17 ± 0.03^**^
Verapamil	Control	−2.45 ± 0.06
	Test concentration 0.1 μM	−1.69 ± 0.05^**^

^**^ P < 0.05; test vs respective control

## Discussion

The findings of the study prove our assumptions that statins may have inhibitory effects on voltage gated calcium channels. These findings can be interpreted in multiple ways. Like, if statins are used in combination with other calcium channel blockers, especially for management of hypertension in obese who may have hypercholestremia where statins may be advised for management of concomitant hypercholestremia. Then what could be the possible effects like additive or synergistic, is yet to be answered on evidence based practice of medicine. As it has been reported earlier that rhabdomyolysis and myositis occur with use of statins and whereas there are reports of upregulation of L type calcium channels in cell lines, hence, this study further confirms that statins inhibit the voltage gated calcium channels. This requires reassessment for dosimetry of statins, particularly, in clinical environments where combination of statins and calcium channel blockers are prescribed together. More, chances of muscles’ weaknesses or rhabdomyolysis should be reassessed in absence and presence of a calcium channel blockers to prove its clinical relevance. More, the use of statins may also be questioned for clinical conditions where maximum doses of statins are recommended. Thus the findings of the studies suggest that simvastatin and fluvastatin shifted the calcium curves to right in relatively in less amount i.e. 4.6 μg/ml and 9.61 μg/ml respectively. While Rosuvastatin and atorvastatin shifted the curves in presence of 6.6 μg/ml and 23.1 μg/ml respectively. Logically speaking, chances of adverse effects should be less with fluvastatin as it shifted the curves to right in relatively high concentration. Nevertheless, the tested statins follow inhibition of voltage gated calcium channels that warrants for reassessment of its dosimetry particularly in presence of other voltage gated calcium channel blockers like verapamil, diltiazem, amlodipine and nifedipine in true clinical environments. Perhaps, reassessing the doses of statins may be helpful to avoid rhabdomyolysis or myositis that mostly appear in shape of adverse effects of the statins rather than to go for its withdrawal or blaming drugs that are already in the market for a good cause.

## Conclusions

Our findings suggest that current statins have relaxant activity on smooth muscles that follow inhibitory effect on voltage gated calcium channels that may explain the possible rationale for gastrointestinal disorders and muscle weakness that is reported with statins.

## Abbreviations

M: Molar
Log10[statin] M: Molar concentration of statin expressed as log10
hrs: Hours
GIT: Gastrointestinal tract
